# A Rigorous 2D–3D Registration Method for a High-Speed Bi-Planar Videoradiography Imaging System

**DOI:** 10.3390/diagnostics14141488

**Published:** 2024-07-11

**Authors:** Shu Zhang, Derek D. Lichti, Gregor Kuntze, Janet L. Ronsky

**Affiliations:** 1 Department of Geomatics Engineering, University of Calgary, 2500 University Dr NW, Calgary, AB T2N 1N4, Canada; ddlichti@ucalgary.ca; 2 Department of Mechanical and Manufacturing Engineering, University of Calgary, 2500 University Dr NW, Calgary, AB T2N 1N4, Canada; gkuntze@ucalgary.ca (G.K.); jlronsky@ucalgary.ca (J.L.R.)

**Keywords:** 2D–3D registration, bundle adjustment, dual fluoroscopy, biplanar videoradiography, magnetic resonance imaging, biomechanics

## Abstract

High-speed biplanar videoradiography can derive the dynamic bony translations and rotations required for joint cartilage contact mechanics to provide insights into the mechanical processes and mechanisms of joint degeneration or pathology. A key challenge is the accurate registration of 3D bone models (from MRI or CT scans) with 2D X-ray image pairs. Marker-based or model-based 2D–3D registration can be performed. The former has higher registration accuracy owing to corresponding marker pairs. The latter avoids bead implantation and uses radiograph intensity or features. A rigorous new method based on projection strategy and least-squares estimation that can be used for both methods is proposed and validated by a 3D-printed bone with implanted beads. The results show that it can achieve greater marker-based registration accuracy than the state-of-the-art RSA method. Model-based registration achieved a 3D reconstruction accuracy of 0.79 mm. Systematic offsets between detected edges in the radiographs and their actual position were observed and modeled to improve the reconstruction accuracy to 0.56 mm (tibia) and 0.64 mm (femur). This method is demonstrated on in vivo data, achieving a registration precision of 0.68 mm (tibia) and 0.60 mm (femur). The proposed method allows the determination of accurate 3D kinematic parameters that can be used to calculate joint cartilage contact mechanics.

## 1. Introduction

Two-dimensional to three-dimensional registration is the process that matches a 3D model of an object with 2D image pairs and recovers the 3D pose of the object. High-speed biplanar videoradiography (HSBV) or dual fluoroscopy (DF) is an X-ray-based imaging system that can capture a series of 2D radiographic image pairs. It uses a low-dose X-ray to provide dynamic imagery of the bones. The 3D bone models can be obtained from magnetic resonance imaging (MRI) or computed tomography (CT) scans. Registering a 3D bone model to sequential 2D image pairs allows the determination of dynamic bony poses and provides valuable 3D kinematics information. Accurately estimating these kinematic parameters is critical for calculating joint cartilage contact mechanics that can provide insights into the mechanical processes and mechanisms of joint degeneration or pathology. For example, with the registered 3D bone models for the tibia (shank bone) and the femur (thigh bone), the tibiofemoral soft tissue model overlap can be quantified to estimate the cartilage deformation or contact regions [1,2,3,4]. An increased cartilage deformation rate under loading could be an early sign of osteoarthritis [2,5]. The cartilage thickness for early osteoarthritic or healthy knees under loading has been found to range from 0.3 mm to 1.2 mm [2]. Therefore, the estimation accuracy of the cartilage contact thickness should be sub-millimeter for an early osteoarthritis diagnosis, and the same accuracy is required for the estimation at registration.

**Figure 1:**
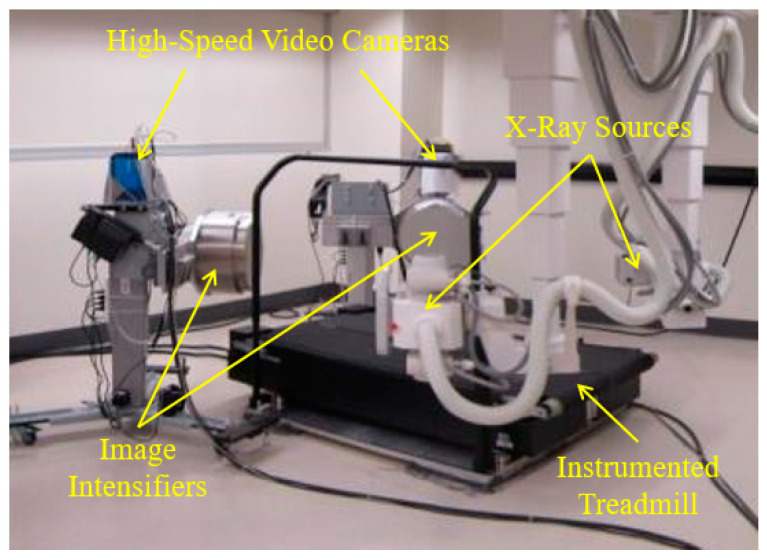
HSBV motion capture system.

The HSBV system in the current study (Figure 1) is located at the Clinical Movement Assessment Laboratory, University of Calgary, Canada. It is composed of two X-ray sources (time-synchronized, G-1086, Varian, Palo Alto, CA, USA), two X-ray image intensifiers (406 mm diameter, E5876SD-P2A, Toshiba, Tokyo, Japan), and two high-frame-rate video cameras (DIMAX, PCO, Kelheim, Germany). Dynamic movement, such as walking or running, is possible with an instrumented treadmill (Bertec, Columbus, OH, USA) positioned within the equipment’s field of view.

The HSBV motion reconstruction procedure has the following four steps: 3D bone model reconstruction, HSBV system photogrammetric self-calibration, biplane videoradiograph series acquisition, and 3D motion reconstruction by 2D–3D registration. As this research focuses on determining cartilage contact, MRI-derived 3D reference bone models are used because they can acquire detailed soft tissue contrast that is difficult to obtain from CT scans. The HSBV system in the current study was calibrated using a bundle adjustment method [6] to obtain the system’s geometry and distortion correction terms. This is a rigorous method and is more flexible than the commonly used direct linear transformation (DLT) technique [6,7].

The 2D–3D registration is the process of determining the six-degree-of-freedom parameters (three translation and three rotation parameters) for the 3D bone model. This can be realized by matching the 3D bone model with the image pair at each frame, which can be conducted by either marker-based or model-based registration.

The Roentgen Stereophotogrammetry Analysis (RSA) system has been well-established for marker-based registration. It can estimate the 3D position of the markers implanted in bones and the kinematics of the skeletal segments [8]. It first estimates the 3D object coordinates from the image pair and then the transformation parameters via Horn’s method [9]. The error from the first estimation is propagated in the second step, so the optimization in the second step is biased. Therefore, there may be more accurate methods for marker-based registration than the RSA method.

There are two commonly used model-based registration methods: intensity-based and feature-based. The intensity-based method matches the intensities of the pixels and voxels between 2D and 3D images and requires digitally reconstructed radiographs (DRRs) generated from 3D CT data. An intensity-based registration accuracy for a dynamic trial was reported [10]. The root mean squared (RMS) errors were 0.88, 0.41, and 0.32 mm in the X-, Y-, and Z-axis for the tibia, and they were 0.60, 0.23, and 0.32 mm for the femur [10]. This is equivalent to the distance of RMS errors of 1.02 mm for the tibia and 0.72 mm for the femur. However, this method is not suitable for MRI data as there is generally no physical correspondence between MRI-based DRRs and radiographs [11,12]. The feature-based method matches the feature locations in 2D and 3D images and minimizes the distances between the corresponding points. A feature-based registration for a dual fluoroscopic imaging system (DFIS), consisting of two C-arms and one treadmill, was reported [13]. The orthogonal images acquired from C-arm are easier for registration. However, the fixed configuration limits the patient’s movement space and, thus, was unsuitable for the free-form HSBV setup in the current study. A modified iterative closest point (ICP) [14] method was used for the 2D–3D registration with the Broyden–Fletcher–Goldfarb–Shanno (BFGS) optimization function in MATLAB. Reference [15] tested the knee kinematics accuracy of a manually flexed-extended cycle from the DFIS system. The difference with the RSA method was 0.24 ± 0.16 mm for the posterior femoral translation and 0.16 ± 0.61° for the internal–external tibial rotation over a flexion-extension cycle [15]. However, this comparison was conducted on the knee joint kinematics, representing a compound effect of the tibia and femur registration. The 3D reconstruction accuracy of the 2D–3D registration was not reported.

A rigid body is a solid body where the distance between any two points on the body remains constant throughout the movement. The rigid-body transformation, with only six parameters, is widely adopted due to its simplicity and stability. However, a non-rigid transformation is also adopted under the following circumstances [13,16]: (1) when distortion or deformation occurs between the data acquisitions; (2) registration for the deformable anatomical structure changes with time, e.g., cardiovascular or tumor; or (3) 3D reconstruction is conducted based on statistical models from a population of subjects. The use of non-rigid transformation for the unchanged bone model has not been seen since a bone is considered a rigid body. However, in a 2D–3D registration, a rigid-body transformation may not be valid if discrepancies exist between the 2D radiographs and the 3D bone model.

The purpose of this study was to develop a 2D–3D registration method that works for both marker-based and model-based registration with high accuracy using MRI-derived bone models and to assess the proposed method’s registration accuracy. The non-rigid transformation needs to be considered and tested to see if it improves registration accuracy. Whereas the model-based registration with submillimeter accuracy is required, as stated previously, marker-based registration is needed to provide the ground truth for the model-based method validation. Therefore, the accuracy requirement of the marker-based registration should be at least three times better than the model-based accuracy.

## 2. Material and Method

### 2.1. Models

#### 2.1.1. Rigid-Body Transformation

A 3D bone model is a set of vertices defined by 3D coordinates to represent the bone. It is also called a point cloud of the bone and can be acquired from an MRI or CT scan. Rigid-body transformation of the 3D bone model can be described by three translational and rotational parameters. For point *i* in the point cloud, with coordinates represented by $\left(X_{i}^{o},Y_{i}^{o},\;Z_{i}^{o}\;\right)$, its transformed coordinates are given as follows:

(1) $$\left[\begin{array}{c} X_{i}\\ Y_{i}\\ Z_{i} \end{array}\right]=M_{\left(\kappa^{t},\varphi^{t},\omega^{t}\right)}\left[\begin{array}{c} X_{i}^{o}\\ Y_{i}^{o}\\ Z_{i}^{o} \end{array}\right]+\left[\begin{array}{c} X^{t}\\ Y^{t}\\ Z^{t} \end{array}\right]$$

where ${(X}^{t},Y^{t},Z^{t})$ are translational parameters; $\left(\kappa^{t},\varphi^{t},\omega^{t}\right)$ are rotational parameters; $\left(X_{i},Y_{i},Z_{i}\right)$ are the transformed model points; and $M_{\left(\kappa^{t},\varphi^{t},\omega^{t}\right)}$ is the rotation matrix that is parameterized by the following Euler angle sequence:

(2) $$M_{\left(\kappa^{t},\varphi^{t},\omega^{t}\right)}=R_{3}(\kappa^{t})R_{2}(\varphi^{t})R_{1}(\omega^{t})$$

where $\left(R_{1},R_{2},R_{3}\right)$ are the rotation matrices about the X, Y, and Z axes, respectively. The point cloud of the 3D bone model can be transformed to match the radiographs using (2). The three translational and three rotational parameters are the unknowns to be determined by the 2D–3D registration.

#### 2.1.2. Non-Rigid Transformation with a Scale

In the current study, the possible sources of discrepancies in the 2D radiographs and the 3D bone model are as follows: (1) errors in the 3D bone model during the segmentation from the CT or MRI scans; (2) potential offsets of the detected 2D features from their actual positions. Therefore, it is necessary to test an appropriate deformation model on the 2D–3D registration to determine whether discrepancies exist.

The following two aspects were considered when choosing the non-rigid transformation model: (1) does it accurately model the deformation so that the registration accuracy can be improved, and (2) will it lead to a higher numerical instability? A higher numerical instability means that the estimated parameters are more sensitive to the initial values or higher correlations are observed between the parameters. A simpler model contains fewer parameters, which usually means less system uncertainty. Thus, the tests of the deformation models should start with a simple model and progressively, if necessary, proceed to more complex models. In medical image registration, the commonly used non-rigid transformation models include scaling, affine transformation, elastic modeling, and freeform deformation. Among these, scaling is the simplest case that only adds a scale factor to the rigid transformation. Thus, the non-rigid transformation with a scale factor was tested in this study. It was applied to the 2D–3D registration and compared with the registration with rigid transformation.

**Figure 2:**
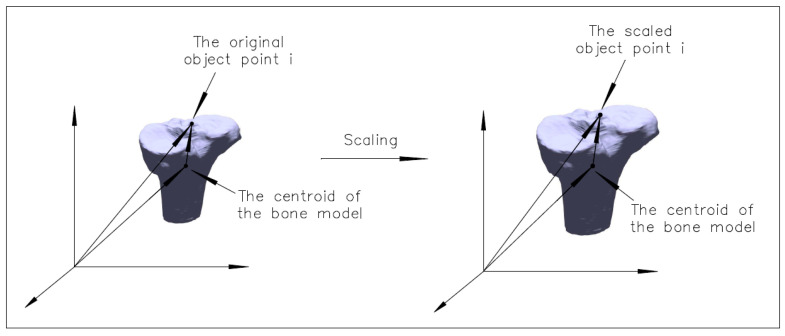
Illustration of a bone model scaling.

Figure 2 illustrates the scaling of the bone model applied in the current study. The object coordinates of the bone model point cloud were reduced to the centroid before being scaled, leaving the centroid of the bone model the same after the scaling. The scaled coordinates ${(X}_{i}^{s},Y_{i}^{s},Z_{i}^{s})$ of the ith model point are as follows:

(3) $$\left[\begin{array}{c} X_{i}^{s}\\ Y_{i}^{s}\\ Z_{i}^{s} \end{array}\right]=\lambda \left[\begin{array}{c} \bar{X}+X_{i}^{o}\\ \bar{Y}+Y_{i}^{o}\\ \bar{Z}+Z_{i}^{o} \end{array}\right]-\left[\begin{array}{c} \bar{X}\\ \bar{Y}\\ \bar{Z} \end{array}\right]$$

where $\left(\bar{X},\bar{Y},\bar{Z}\right)$ is the centroid of the object point cloud; ${(X}_{i}^{o},Y_{i}^{o},Z_{i}^{o})$ are the original coordinates of the model point i; and λ is the scale factor applied to the coordinates of the object points. If no scale exists, the scale factor is one; that is, the rigid transformation has a scale factor of one. By combining the rigid transformation (1) and the scaling (3), the non-rigid transformation with a scale factor is given as follows:

(4) $$\left[\begin{array}{c} X_{i}\\ Y_{i}\\ Z_{i} \end{array}\right]=M_{\left(\kappa^{t},\varphi^{t},\omega^{t}\right)}\left((\lambda -1)\left[\begin{array}{c} \bar{X}\\ \bar{Y}\\ \bar{Z} \end{array}\right]+\lambda \cdot \left[\begin{array}{c} X_{i}^{o}\\ Y_{i}^{o}\\ Z_{i}^{o} \end{array}\right]\right)+\left[\begin{array}{c} X^{t}\\ Y^{t}\\ Z^{t} \end{array}\right]$$

#### 2.1.3. Coordinate Systems and Collinearity Equations

**Figure 3:**
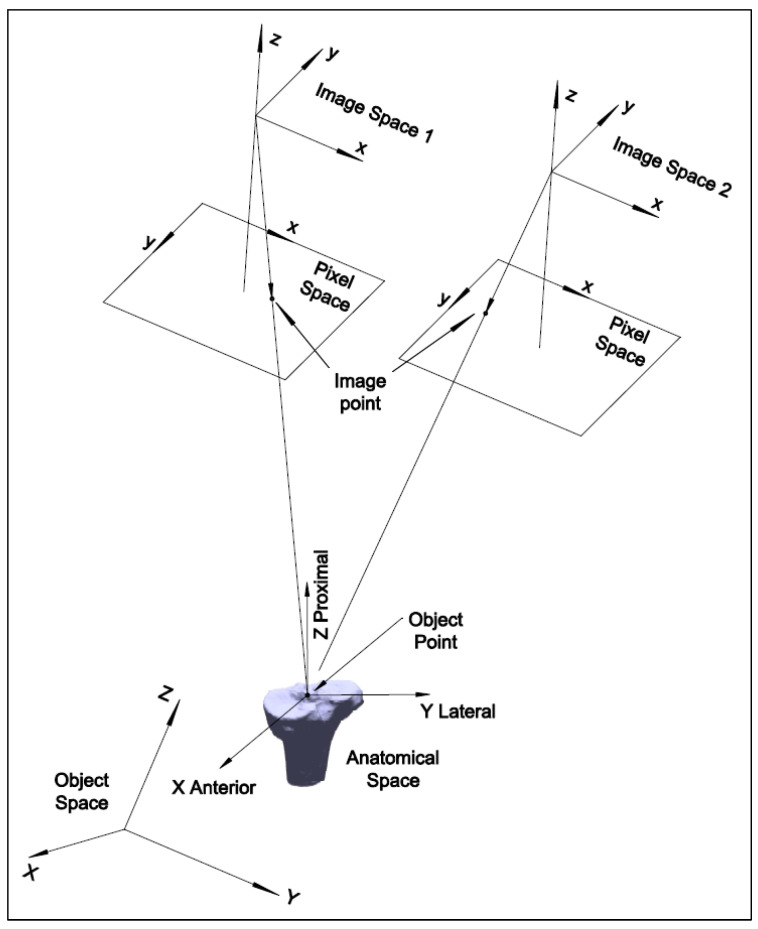
Schematic of the registration coordinates systems.

The HSBV system uses different coordinate systems, including anatomical space, object space, image space, and pixel space (Figure 3). The 3D anatomical space has the physical significance of the subject of interest. It is used for kinematic studies. The 3D object space and 3D image space are defined by the HSBV system geometry acquired from the calibration. The pixel space is the 2D image coordinate system.

The pinhole camera model with ‘lumped components’, including the X-ray source, the image intensifier, and the video camera, is used for the HSBV system. The collinearity model describes the condition of the perspective center of the imaging system, the image point, and its corresponding object point, which lies in a straight line. Figure 3 also shows the geometry of the collinearity condition for an image pair in the HSBV system. The collinearity equations are the key to establishing a relationship between the 3D object points and the 2D image points, which can be represented as follows:

(5) $$\begin{array}{c} x_{i}=x_{p}-c\frac{U_{i}}{W_{i}}+∆x_{i}\\ y_{i}=y_{p}-c\frac{V_{i}}{W_{i}}+∆y_{i} \end{array}$$

where $\left(x_{i},y_{i}\right)$ are the image coordinates of the *i*th point; c is the principal distance; $\left(x_{p},y_{p}\right)$ are principal point coordinates; $\left(∆x_{i},∆y_{i}\right)$ are the image distortion correction terms; ${(U}_{i},V_{i},W_{i})$ are image space coordinates transformed from the object space; this transformation is given as follows:

(6) $$\left[\begin{array}{c} U_{i}\\ V_{i}\\ W_{i} \end{array}\right]=M_{\left(\kappa ,\varphi ,\omega \right)}\left[\begin{array}{c} X_{i}-X^{C}\\ Y_{i}-Y^{C}\\ Z_{i}-Z^{C} \end{array}\right]$$

where $\left(X^{C},Y^{C},Z^{C}\right)$ are perspective center coordinates in object space; ${(X}_{i},Y_{i},Z_{i})$ are transformed model points in the object space acquired from (1) or (4); and $M_{\left(\kappa ,\varphi ,\omega \right)}$ is the rotation matrix from the object space to the image space. The image distortion terms $\left(∆x_{i},∆y_{i}\right)$ in (5) are provided by the system calibration, along with the estimation of the exterior orientation parameters (EOPs, $X^{C},Y^{C},Z^{C},\kappa ,\varphi ,\omega$) and the interior orientation parameters (IOPs, $x_{p},y_{p},c$) of each video camera. Additional details about the sensor model EOPs and IOPs can be found in [6].

### 2.2. Marker-Based 2D–3D Registration

#### 2.2.1. Target Extraction

**Figure 4:**
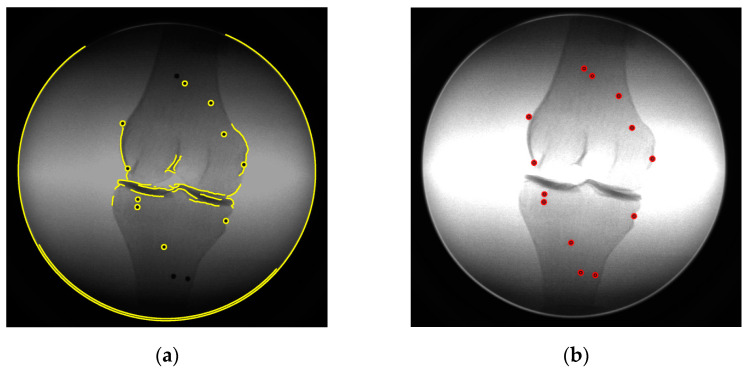
Target-extracting illustration; (**a**) edge detected from the image; (**b**) complete targets extracted. Yellow lines: detected edges, red lines: target ellipses, and red dots: target centers.

Marker-based registrations require corresponding coordinates for the markers in both the image and object spaces. Thus, acquiring accurate image coordinates of the marker centers through target extraction is essential. In this research, the Canny edge detection [17] is used to first extract the edges from the images, which potentially contain the marker edges (Figure 4a). Then, ellipse fitting [18,19] is used to estimate the centers of the markers from the extracted edge points. After all the connected components are fitted to the ellipses, irrelevant ellipses may exist due to the outliers from the edge detection. Since the size of the markers is similar, the semi-major and semi-minor axis of the marker ellipses should be within a specific range. The irrelevant ellipse(s) can be removed by constraining the semi-axis length. It is possible that not all the targets were detected because no edge points were found (for example, two markers, as shown in Figure 4a. These non-identified targets were in the low-intensity area and were not detected by Canny edge detection due to the low contrast between the beads and the surrounding area. This problem can be solved by image enhancement. After the image intensity is increased, the contrast of the dark area is enlarged, and then the edges can be detected. Figure 4b shows the markers extracted from the image with increased intensity.

#### 2.2.2. Transformation Parameters Estimation

The transformation parameter estimation can be achieved by back-projection (optimizing in 3D) or projection (optimizing in 2D). In this paper, a projection method is proposed because it is a one-step method that it does not require an assumption to back-project the edge points to their 3D form. The assumption is that the point’s depth is the same as its matching model point.

An automatic initialization method with 64 starting poses [20] is used to acquire the initial transformation prior to the estimation. The goal of the initialization is to determine the approximate translation and rotation parameters that are within the capture range for the registration with reasonable computation time. In the 2D–3D registration, the rotation parameters are sensitive to the initial values because they cannot overcome a rotation of more than a certain range. To address this problem, the search space can be evenly subdivided. Based on the testing results [20], a reliable initialization procedure was constructed. Starting from 64 poses, the 2D–3D registration for each starting pose was performed. The one with the smallest root mean square error (RMSE) from registration was considered the best coarse approximation of the registration. This method does not need any prior knowledge regarding the pose or parameterizing.

**Figure 5:**
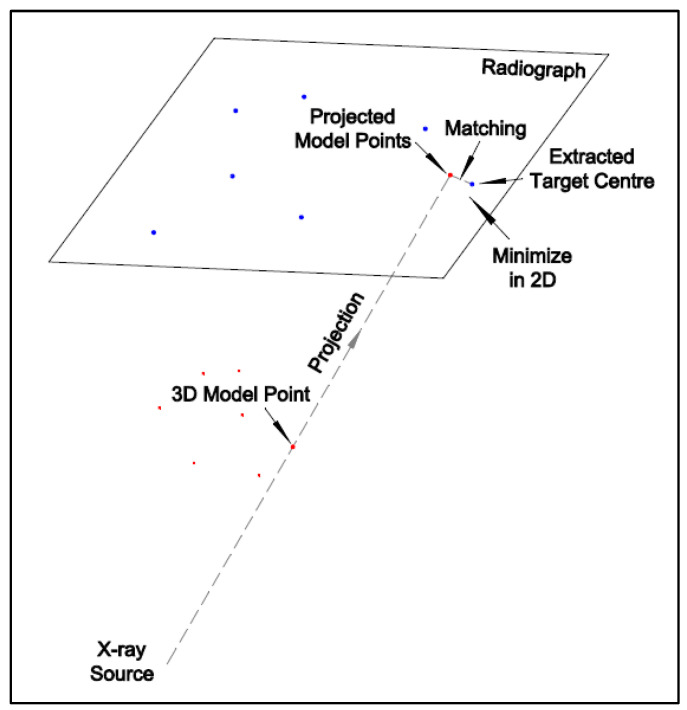
Illustration of the projection of the model points and the 2D distance minimization (Red dot: 3D model point and its projected point; blue dot: extracted target center).

**Figure 6:**
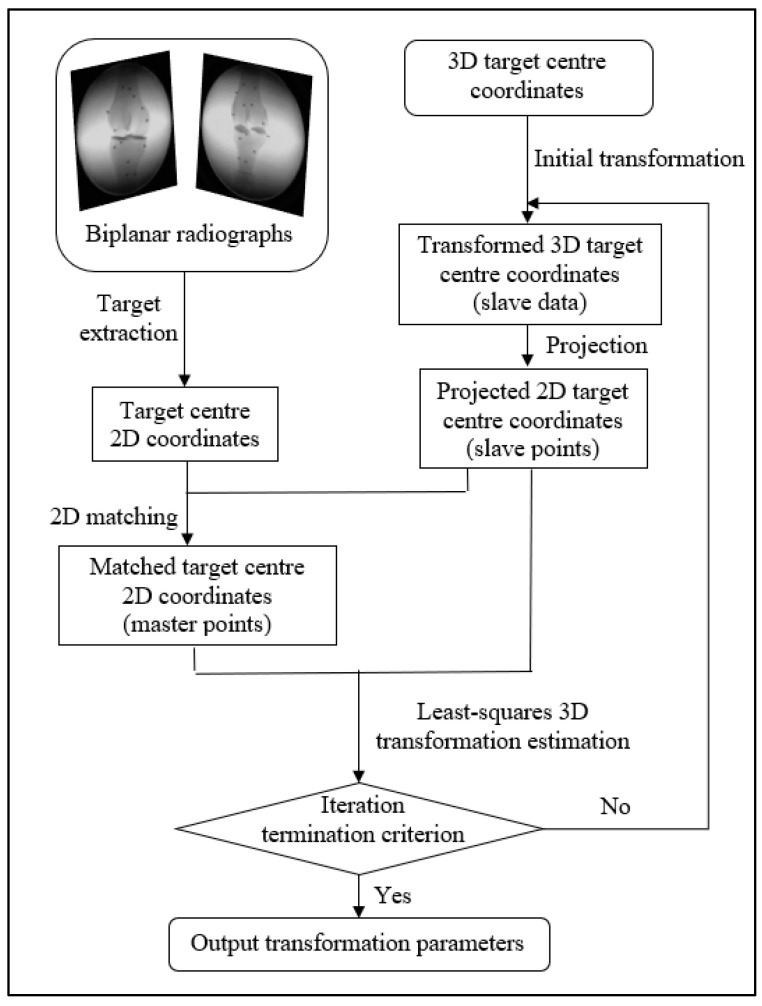
Flow chart of the marker-based registration.

After the initial transformation, the 3D model coordinates of the target centers are projected onto the 2D image plane using (5) and (6). The nearest point for each projected point can be found in the target centers extracted from the image. The one-to-one correspondence can be automatically aligned based on the point-to-point distance [20]. By minimizing the 2D distances between the target extractions (master data) and the projected 2D model points (slave data), the 3D transformation can be calculated directly. Figure 5 shows the projection of the model points and the 2D distance minimization. Figure 6 illustrates the flow chart of the projection method. The least-squares method is used to estimate the transformation parameters, which are given as follows:

(7) $$\sum_{i=1}^{N}{\left\|\left[\begin{array}{c} x_{i}^{p}\\ y_{i}^{p} \end{array}\right]-\left[\begin{array}{c} x_{i}^{m}\\ y_{i}^{m} \end{array}\right]\right\|}^{2}\to min$$

where $\left(x_{i}^{p},y_{i}^{p}\right)$ are the projected coordinates of the *i*th model point (slave point); $\left(x_{i}^{m},y_{i}^{m}\right)$ are extracted image coordinates of the *i*th target center, that is, the master point; and *N* is the total number of beads.

The projected coordinates can be acquired from the 3D model point coordinates after using Equation (1) for transformation and Equations (5) and (6) for projection. Both procedures involve Cardan angles with complex trigonometric functions and the risk of gimbal lock problems, which makes it challenging to compute the design matrix. The unit quaternions were used to represent the 3D rotations in the transformation procedure. Then, the rotation matrix in the rigid-body transformation of Equation (1) was substituted by Equation (5). In this case, the design matrix was as follows:

(8) $$A=\frac{\partial l}{\partial x}=\left(\begin{array}{ccccccc} \vdots & \vdots & \vdots & \vdots & \vdots & \vdots & \vdots \\ \frac{\partial f_{x_{i}}}{\partial X^{t}} & \frac{\partial f_{x_{i}}}{\partial Y^{t}} & \frac{\partial f_{x_{i}}}{\partial Z^{t}} & \frac{\partial f_{x_{i}}}{\partial q_{0}} & \frac{\partial f_{x_{i}}}{\partial q_{1}} & \frac{\partial f_{x_{i}}}{\partial q_{2}} & \frac{\partial f_{x_{i}}}{\partial q_{3}}\\ \frac{\partial f_{y_{i}}}{\partial X^{t}} & \frac{\partial f_{y_{i}}}{\partial Y^{t}} & \frac{\partial f_{y_{i}}}{\partial Z^{t}} & \frac{\partial f_{y_{i}}}{\partial q_{0}} & \frac{\partial f_{y_{i}}}{\partial q_{1}} & \frac{\partial f_{y_{i}}}{\partial q_{2}} & \frac{\partial f_{y_{i}}}{\partial q_{3}}\\ \vdots & \vdots & \vdots & \vdots & \vdots & \vdots & \vdots \\ 0 & 0 & 0 & \frac{\partial g}{\partial q_{0}} & \frac{\partial g}{\partial q_{1}} & \frac{\partial g}{\partial q_{2}} & \frac{\partial g}{\partial q_{3}} \end{array}\right)$$

where $g\left(x\right)=q_{0}^{2}+q_{1}^{2}+q_{2}^{2}+q_{3}^{2}=1$. It is used to constrain the unit quaternion condition.

The projection method can directly estimate the 3D transformation parameters, the three translational parameters, and the three rotational parameters in Equation (1) by minimizing the 2D distances using the non-linear least-squares estimation (7). The whole projection procedure, including 2D matching and 3D transformation, is repeated until the termination criterion is met. To exclude the outliers, matching pairs whose distance is more than three times the standard deviation are considered mismatches and are rejected.

### 2.3. Model-Based 2D–3D Registration

**Figure 7:**
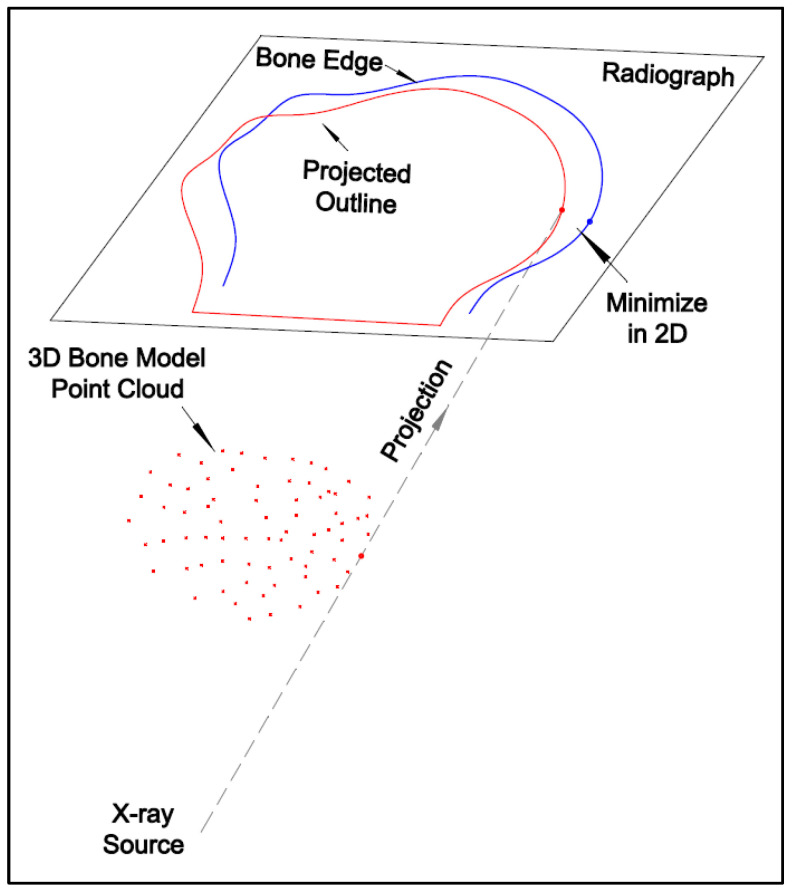
Illustration of the model-based registration.

The development of marker-based registration methods provides the mathematical basis for model-based registration. The transformation estimation method introduced in the previous section can be adapted into the model-based registration procedure, as shown in Figure 7.

**Figure 8:**
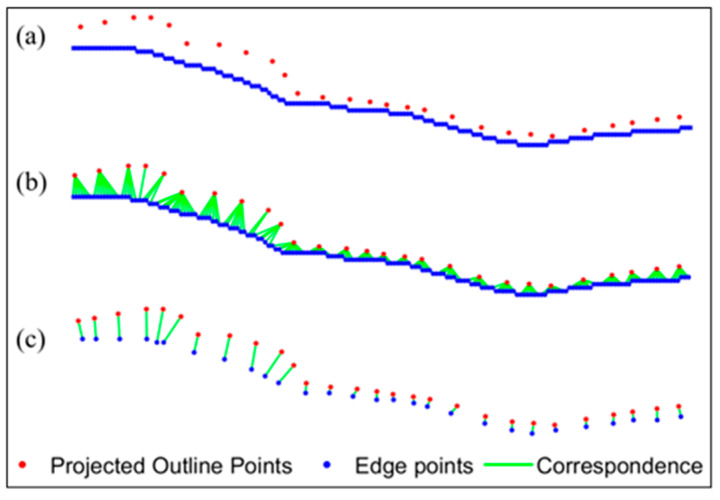
Projected outline point and edge point matching. (**a**) Points before matching, (**b**) matching from each edge point, and (**c**) one-to-one correspondence.

Instead of extracting target centers for the marker-based registration, the feature-based registration uses the edge points of the subject. The multi-threshold Canny edge detector [20] is used to acquire the edge points of the bones from the HSBV image pairs. The radiographs depict the internal view of an object because the materials that comprise the object attenuate the X-ray differently depending on their densities and structures. The variation in contrast across the X-ray image is typically lower than the optical image, especially for the knee joint comprised bones and cartilage. Detecting the edge of steel beads may be easy since metal absorbs most radiation and provides a high contrast. But it is challenging to detect the bone edges from the radiographs. As such, the Canny detector parameters need to be adjusted carefully to acquire better edge points. Thus, a multi-thresholding strategy that combines multiple Canny edge detectors is adopted. After the empirical testing with a set radiograph, a bank of Canny edge detectors with 25 filters can be developed. By overlaying the edges detected by all the filters, the edge points are more robust than those acquired from only one Canny detector. The overlain edges are further thinned based on their connectivity because there might be multiple responses to a single edge. Those edge points are the master dataset for registration. The slave points are acquired from the 3D bone model point clouds. The 3D model points are first projected onto the 2D image plane using Equations (3)–(5). Then, the outline points of the 2D model points are extracted. These outline points are the slave dataset for the registration. With the 2D edge points and the 2D outline points shown in Figure 8a, one-to-one correspondence is required. The method to establish correspondence is based on point-to-point distance [20]. The nearest outline point can be found from each edge point. While an outline point may be matched to a few edge points (Figure 8b), only the one with the shortest distance is kept so that the one-to-one correspondence is preserved (Figure 8c). Finally, the least-squares estimation described for the marker-based registration is used to determine the transformation parameters.

## 3. Experiments and Validation Tests

### 3.1. Validation Using the 3D-Printed Bone Model

The model-based 2D–3D registration is a modern technique that enables non-invasive 3D motion capture using an HSBV system. However, ground truth is not available for the registration, which means that the accuracy of the registration cannot be quantified. Therefore, solutions with a high accuracy obtained from the marker-based registration are used as the ground truth for validating the model-based method.

#### 3.1.1. Experiment

**Figure 9:**
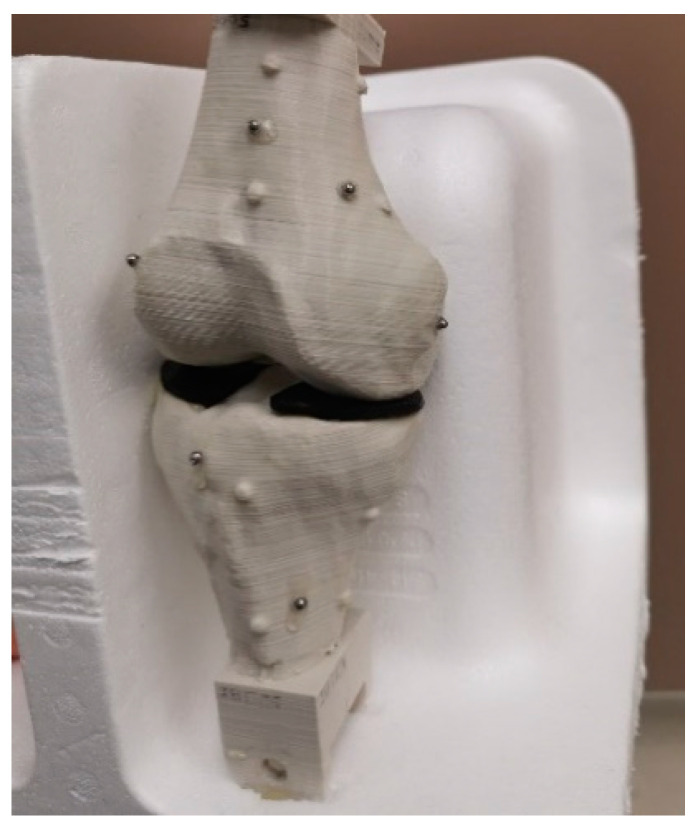
The 3D-printed knee joint used for the validation procedure.

The 3D-printed bone: A 3D-printed human knee joint (Figure 9) was used in this research. The tibia and femur were 3D-printed by the MakerBot Replicator Z18 printer using polylactic acid. The original 3D bone model files used for printing were from a CT segmentation of a healthy human knee. As shown in Figure 9, black rubber was inserted between the bones to represent the cartilage because this material had a similar X-ray attenuation to cartilage. It was also used to glue the 3D-printed tibia and femur together. Steel beads (3 mm diameter) were glued to the surface of the bones. Six and seven beads are randomly distributed over the bone surface of the tibia and femur, respectively. In contrast with the previous literature that only uses three beads for each bone [10,15], six to seven beads can provide more redundancy and secure higher accuracy.

CT scan and segmentation: The bone model file used for 3D printing was constructed from the CT scan (Revolution GSI, GE HealthCare, Chicago, IL, USA). The slice thickness was 0.625 mm, and the voxel size was 0.2148 × 0.2148 × 0.625 mm. Afterward, automatic segmentation was performed to obtain the bone model point cloud as well as the 3D coordinates of the fiducial markers (beads) using the Amira software 2019 (Thermo Fisher Scientific, Waltham, MA, USA).

**Figure 10:**
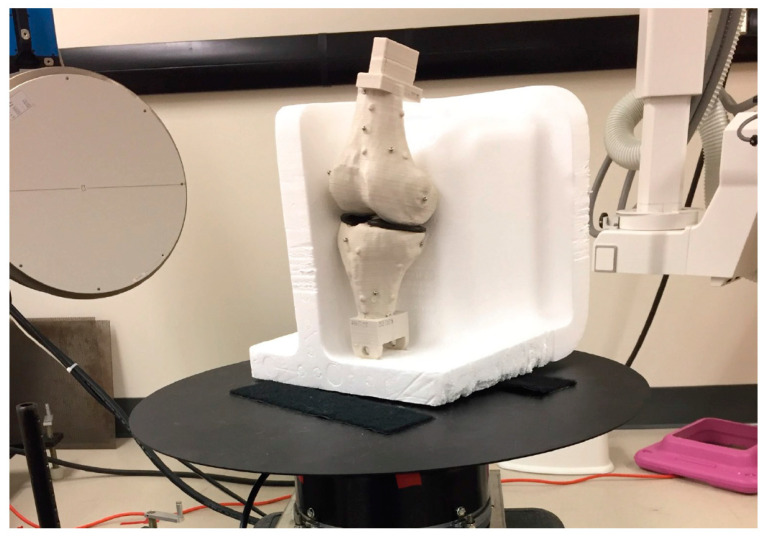
The 3D printed bone models on the turntable for HSBV imaging. (copyright, Luis Alonso Figueroa).

Biplanar radiograph imaging: The 3D printed bone model was imaged using the HSBV system with the configuration shown in Figure 10 at 12 Hz. There were four repeat trials captured for the axial rotation. There were discontinuities between the trials because the turntable was not completely stopped right after the video camera paused recording. Thus, the turntable would be over-rotated from its last “recorded” position when the new trial began.

**Figure 11:**
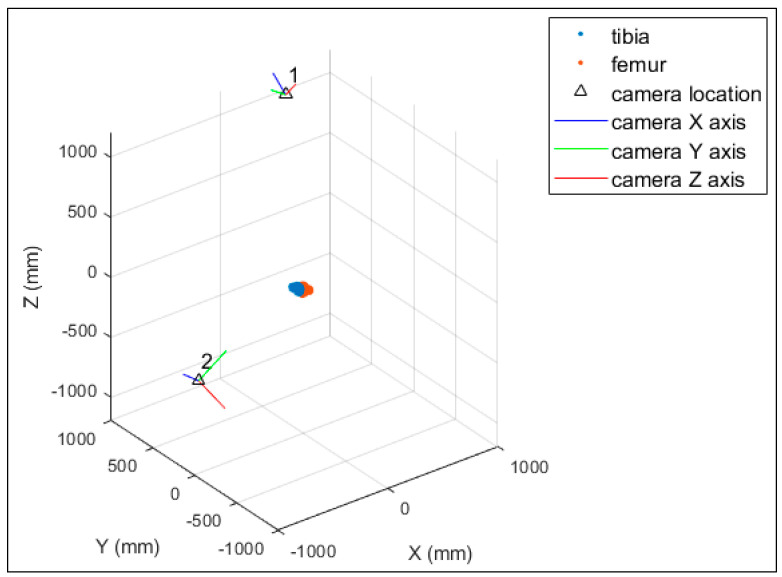
The system geometry with the selected EOPs for the 3D–printed bone model experiment.

Figure 11 illustrates the calibrated system geometry in the object space with the selected pair of EOPs and the point clouds of the 3D-printed bone models. The object space coordinate system is of no great significance for each axis. However, the bones and the cameras can be used as reference objects. Thus, it can be considered that the X-axis lies mainly in the vertical direction, the Y-axis lies mainly in the depth direction, and the Z-axis lies mainly in the horizontal direction.

Reference bone model validation by FaroArm: A validation test on the 3D reference bone models was conducted to confirm if they matched the actual 3D printed bones. The 3D coordinate measurement machine (CMM) FaroArm Platinum (FARO, Lake Mary, FL, USA) was used to collect the point cloud of the 3D-printed bones for the femur and tibia. The precision of the FaroArm was 0.025 mm, as determined by the single-point articulation performance test. The point clouds were collected manually by moving the arm freely and pointing the probe onto the surfaces of the 3D-printed bones. There were 456 points collected for the femur and 307 points for the tibia. The point cloud of the 3D-printed bones acquired from the FaroArm was compared with the reference bone models to check for a scaling problem.

#### 3.1.2. Accuracy Assessment and Validation

The marker-based registration was assessed by comparing the matching points’ RMS errors in the pixels of the proposed method with the RSA method. Since the beads’ 3D coordinates and their 2D image coordinates have fixed correspondence, this can be considered an estimate of the registration accuracy. The mean precision of the registration parameters of the proposed method was also checked.

The accuracy of the model-based registration can be quantified by comparing the model-based registration results to the ground truth, that is, the marker-based registration. The accuracy assessment was performed in two approaches. The first approach assessed the accuracy of the registration parameters. The difference between the two sets of parameters showed how much the object transformed from the model-based registration position to the ground truth. The second approach assessed the 3D reconstruction accuracy of the registration. This accuracy was acquired by comparing the bead coordinates from the model-based registration to the marker-based registration. The Euclidean distance of the marker positions between the model- and marker-based tracking measured the 3D tracking accuracy of the model-based registration independent of the choice of coordinate system.

The model-based registration with rigid and non-rigid transformations was compared. If the accuracy of both methods was similar, there were no scale discrepancies between the 2D data and the 3D bone models. Then, the rigid-body transformation was appropriate for the registration. However, if the accuracy of the non-rigid transformation estimation was higher than the rigid-body transformation, the discrepancies between the 2D and 3D data were evidenced. In this case, the discrepancies need to be investigated from the 3D bone models, the 2D data, or both. The point clouds acquired from the FaroArm were used to verify the 3D bone model segmented from the CT scan. Also, edges detected from the 2D radiographs were examined to investigate if they coincided with the actual edge of the bones.

### 3.2. Experiment Using the In-Vivo Knee Joint

#### 3.2.1. Experiment

The turntable experiment acquired ground truth from the marker-based registration to determine the accuracy of the model-based 2D–3D registration. However, it was a simulated experiment that used the 3D-printed bone model as a replica of real knee bones. Also, the movement of the bone was primarily rotation about the vertical axis, whereas the translations and rotations for the other two axes were negligible. Thus, it was necessary to test the methods using in vivo data during a dynamic movement. In this experiment, the registration accuracy could not be acquired due to no ground truth. However, the registration precision could be obtained so that the methods proposed in the current study were validated by in vivo data.

MRI scan and segmentation: The MRI scan of the right knee of a healthy participant was acquired using a high-resolution MRI system (3 Tesla Discovery 750, GE HealthCare, Chicago, IL, USA). The scan parameters were as follows: repetition time: 7.513 ms; echo time: 2.32 ms; imaging frequency: 127.7671 MHz; flip angle: 35°; spacing between slice: 0.5 mm; slice thickness: 1 mm; slice numbers: 204; pixel spacing: 0.3516 mm × 0.3516 mm; and rows and columns: 512 × 512. The MRI images were manually segmented in the Amira software 2019 to acquire the 3D bone models of the tibia and the femur. The cartilage models were also segmented for soft tissue analysis.

**Figure 12:**
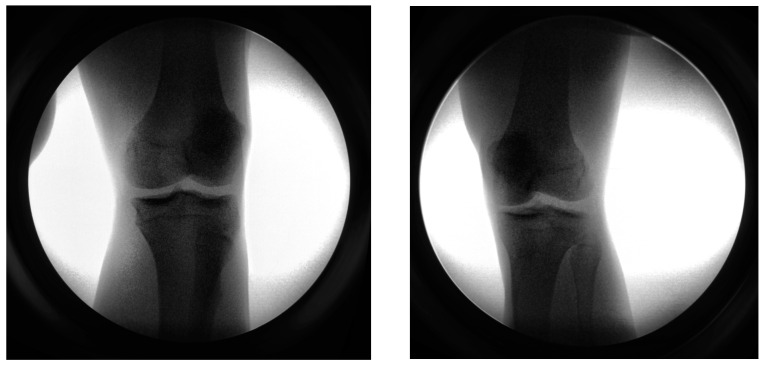
A sample image pair for in vivo knee joint testing.

Biplanar radiograph imaging: The HSBV image pairs of a participant’s right knee were acquired at the Clinical Movement Assessment Laboratory, University of Calgary. Biplanar videoradiography images were collected at 20 Hz while the patient was walking on the treadmill. The radiographs were taken at a resolution of 2016 by 2016 pixels. A sample image pair is shown in Figure 12.

**Figure 13:**
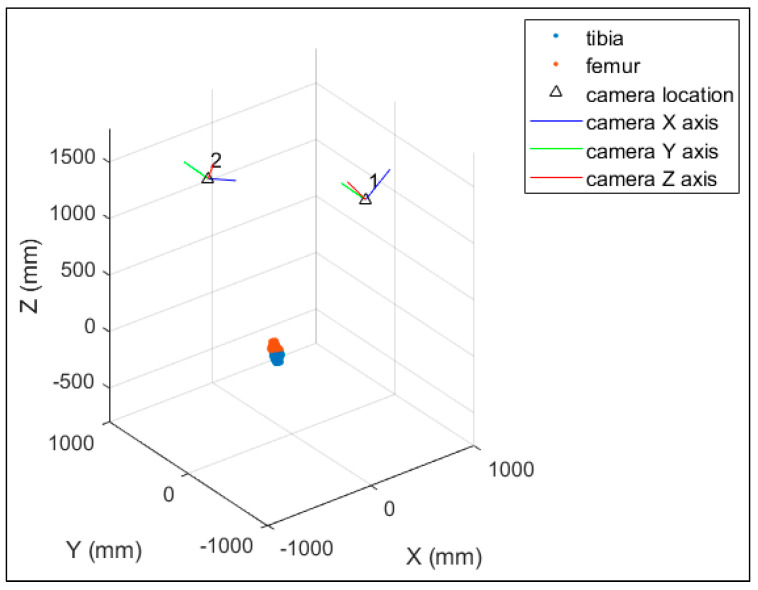
Calibrated system geometry showing the 3D bone models (tibia in blue and femur in orange) and the camera locations for the in vivo experiment.

Figure 13 illustrates the calibrated system geometry in the object space and the point clouds of the 3D subject-specific bone models. As with the printed bone model experiment, the object space coordinate system is of no great significance for each axis. However, the bones and the cameras can be used as reference objects. Thus, it can be considered that the X-axis lies in the horizontal direction, the Y-axis lies mainly in the vertical direction, and the Z-axis lies mainly in the depth direction.

#### 3.2.2. Precision Assessment

The precision assessment was performed for 89 consecutive image pairs. Since a direct comparison of the registration parameters is not available, the registration precision was measured by the RMS distance between the registered 3D bone model points and the back-projected edge points [20]. The edge points were back-projected into the 3D object space by assuming that the point’s depth was the same as its matching model point [20]. The rigid and non-rigid transformations were also applied to the registration and compared.

## 4. Result and Discussion

### 4.1. Results from the 3D-Printed Bone Model

#### 4.1.1. Marker-Based Registration Results

Table 1 lists the RMS of the residuals of all epochs of the RSA method and the proposed method. The proposed method improves the registration accuracy from the RSA method by 3.5% for the tibia and 4.7% for the femur. The RSA method has higher residuals than the proposed method because the RSA method has two separate estimation processes. It first estimates the 3D object coordinates from the image pair and then the transformation parameters by Horn’s transformation. The error from the first estimation is propagated into the second step, so the optimization in the second step is biased. In contrast, the proposed method performs a single-step estimation to optimize the unknown parameters so that all the errors are considered at once. This improvement is a result of the rigorous, one-step estimation that directly minimizes the matching distances in 2D.

The registration parameters’ mean precision from the proposed method is shown in Table 2. For the tibia, the registration precision is 0.06–0.12 mm for the translational parameters and 0.09–0.10° for the rotational parameters. For the femur, the registration precision is 0.05–0.07 mm for the translational parameters and 0.08–0.09° for the rotational parameters. The registration precision of the femur is higher than the tibia in all parameters because the femur has seven beads versus the six of the tibia.

#### 4.1.2. Model-Based Registration Results

Rigid registration accuracy: Table 3 provides the registration accuracy determined by comparing the model-based registration results to the ground truth obtained from the marker-based registration for all epochs. For the translational parameters, the bias is up to 0.32 mm, and the standard deviation is up to ±0.61 mm. For the rotational parameters, the bias is up to 0.34°, and the standard deviation is up to ±1.22°. The bias and the standard deviation of the *ω* rotation are higher than the other two rotational parameters, especially for the femur. The *ω* rotation represents the rotation about the X-axis, which closely corresponds to the vertical axis in this case. Due to the nearly symmetric bone shape, it is hard to determine the proper alignment, especially for the femur, which means that rotating the bone about the vertical axis within a small range can still obtain a similar fit.

**Figure 14:**
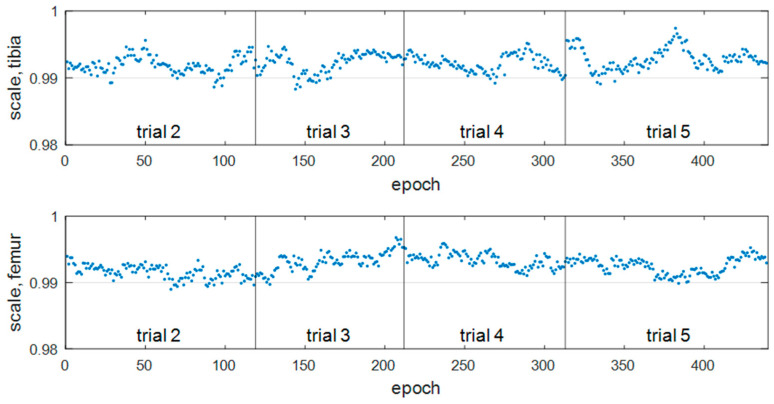
Scale factors λ estimated per epoch with non-rigid registration.

Non-rigid registration accuracy: The non-rigid transformation with a scale factor was tested for the model-based registration. Figure 14 shows the estimated scale factor in all the epochs. Table 4 provides the mean value and standard deviation of the estimated scale factors within each trial and all the epochs. Since the scale factors among different trials are relatively close, a constant scale factor was used to obtain the stable model-based registration results, that is, 0.9923 for the tibia and 0.9926 for the femur.

**Figure 15:**
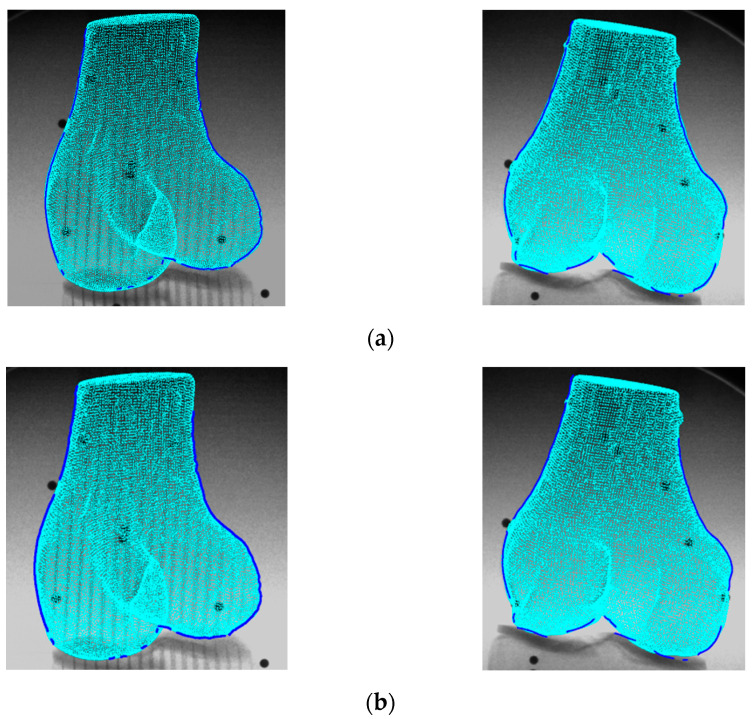
Rigid (**a**) and non-rigid (**b**) registration comparison of the femur for an image pair.

Figure 15 illustrates the femur’s rigid and non-rigid registration comparison for a single frame. It can be observed from the rigid registration result that there are offsets between the bone edges and the outline of the projected bone model point cloud. These offsets are primarily reduced in the non-rigid registration results, where the edges and the bone models match better in both horizontal and vertical directions.

Table 5 provides the registration accuracy after introducing the scale factors. The improvements gained by non-rigid transformation are 4–52% for the translational parameters and 18–56% for the rotational parameters. Compared with the rigid transformation, the registration accuracy was significantly improved. The Z translation accuracy for both the tibia and femur was mainly improved because the scale error was correctly modeled. Since the 2D and 3D data were better matched, the errors in the X translation of the tibia due to lack of edges were reduced. Likewise, the errors in the ω rotation of the femur due to the bone shape were also reduced.

Table 6 provides the 3D reconstruction accuracy comparison between the rigid and non-rigid registration. This was determined by comparing the registered bead coordinates from the model-based registration to the ground truth (bead coordinates from marker-based registration). The improvements gained by non-rigid transformation were 16–33% for the tibia and 0–20% for the femur.

#### 4.1.3. Non-Rigid Transformation Validation Results

**Figure 16:**
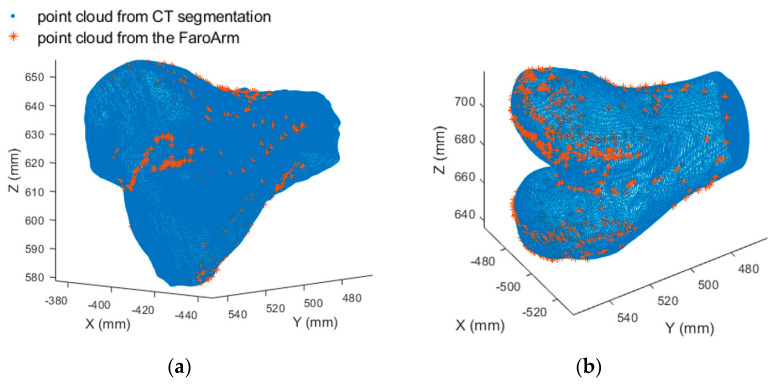
Illustration of bone model point clouds 3D–3D registration: (**a**) tibia; (**b**) femur.

The validation of the 3D bone model: The non-rigid registration with a scale factor suggests improved accuracy over the rigid transformation model. The alignment of the point clouds from FaroArm to the 3D bone model is shown in Figure 16. Both rigid registration and non-rigid registration with a scale factor were performed for the 3D–3D registration. The registration precision was the RMS of the 3D matching distances. The precision difference between the rigid and non-rigid transformation was 0.006 mm for the femur and 0.008 mm for the tibia. The precision change was insignificant, indicating no scale error due to the 3D bone model. The estimated scale factors were 1.0025 for the femur and 1.0032 for the tibia, suggesting that the bone model from the CT segmentation was slightly larger than its actual size. This is the opposite of the scale factors acquired from 2D–3D registration, which were smaller than one. Thus, it can be concluded that the CT segmentation is not the source of the scaling problem.

**Figure 17:**
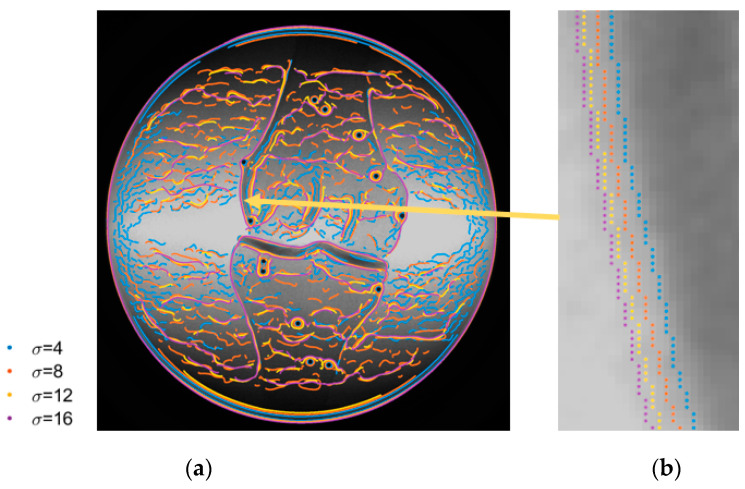
Canny edge detection with different σ values of a sample image: (**a**) full image; (**b**) zoomed-in area.

Figure 17a shows the edge detected by the Canny edge detector with the standard deviation of the Gaussian operator, σ, and values set to 4, 8, 12, and 16, respectively. The zoomed-in area in Figure 17b shows that the offsets exist between the edges detected with different σ values. The multi-thresholding edge detection strategy in the current study used the σ value ranges from 4 to 8 to acquire more complete edges for the registration. The edges from σ value at 4 and 8 lie on the inner side of the bone edge, which coincides with Figure 16, where the edges lie on the inner side of the registered bone model. Thus, it can be concluded that the discrepancies between the 2D and 3D data are due to edge detection.

Whereas it is necessary to use the different σ values to obtain more complete bone edges, it is not ideal for modeling the discrepancies with a single scale factor because the offsets vary with different σ values. However, more complicated deformation models require more parameters, e.g., elastic and freeform deformation. They may lead to higher numerical instability, meaning that the estimated parameters are more sensitive to the initial values and have higher correlations. Therefore, the non-rigid registration with a scale factor was chosen in the current study to achieve improved registration accuracy.

### 4.2. Results from the In-Vivo Knee Joint Experiment

**Figure 18:**
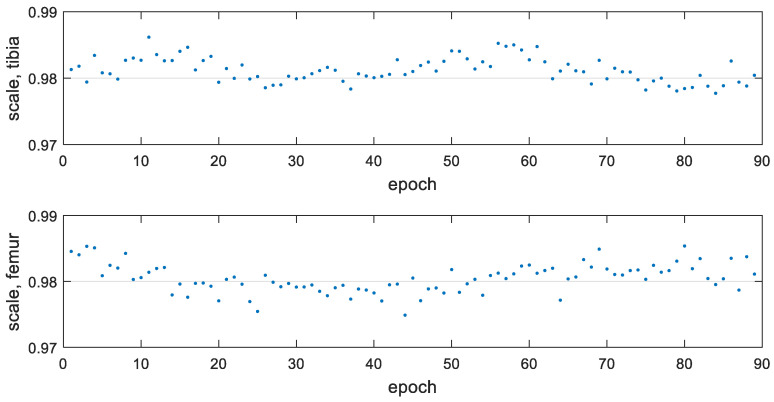
Scale factor λ estimated per epoch for the in vivo experiment with the non-rigid registration.

Figure 18 shows the scale factors estimated in each epoch for the non-rigid transformation. The average estimated scale was 0.9812 for the tibia and 0.9805 for the femur. These values are smaller than 0.9923 (tibia) and 0.9926 (femur) estimated for the 3D-printed bone model in Section 4.1.2. The difference in scale factors may be due to the different materials of the imaged bone models. The constant scale factors were applied to the tibia and femur, respectively. The registration precision comparison between the rigid and non-rigid transformation is shown in Table 7. With the non-rigid transformation, the 3D reconstruction errors for the tibia were reduced from 0.93 mm to 0.68 mm, with an improvement of 27%. The same errors for the femur dropped to 0.60 mm from 0.88 mm, which is 32% better as a percentage. Compared with a previous work [10] that obtained the distance RMS errors of 1.02 mm for the tibia and 0.72 mm for the femur, it was 33% better for the tibia and 17% better for the femur.

## 5. Discussion and Conclusions

This paper presents a complete 2D–3D registration solution with an accuracy assessment for the HSBV system suitable for MRI-based bone models. It is a rigorous method that provides accurate results for both marker-based and model-based registration. Compared with the state-of-the-art methods, the main contributions of this work are as follows:

1. A rigorous one-step projection method is proposed. It works for both marker-based registration and model-based registration with high efficiency. It is an improvement from the previous back-projection method because it does not need the extra step to back-project the matching image coordinates into their 3D form.
2. The discrepancy in the 2D–3D registration was validated for the first time. It was tested by adding a scale factor to the transformation model. The 3D reconstruction accuracy improved by 29% (tibia) and 16% (femur) in the 3D printed bone model experiment, whereas it improved by 27% (tibia) and 32% (femur) in the in vivo knee experiment. This discrepancy is demonstrated to be caused by the offset between the detected edge points in the radiographs and their actual position.
3. A 3D-printed bone model with inserted beads was used to validate the proposed method. The results indicate that the proposed model-based 2D–3D registration method can provide sub-millimeter accuracy with high efficiency.

Despite the success of this approach, there are many future avenues of work to be pursued.

1. Edge detection improvement. The Canny edge detection used in the current study cannot obtain the complete edges at the low contrast area, especially for the contact area overlapping with the cartilage. Missing edges at the contact surface deteriorate the registration accuracy along the vertical axis. Thus, the registration accuracy could be further improved if more edges are acquired. Since the parametrizing of the Canny edge detection was tested extensively, it is worth trying a different edge detection strategy, e.g., deep learning-based edge detection. Deep learning may be used to increase the accuracy of edge detection and outlier removal [21]. The image quality will also affect the edge detection and registration accuracy, but we have not conducted any tests to quantify this. However, image quality is expected to increase due to new solid-state X-ray recording devices with higher spatial resolution.
2. Fully automated registration. In spite of the effort to automate the registration procedure, this method might still need user intervention at some point. The method automatically validates the registration results in each epoch with a registration objective function. However, the edges need to be visually checked when the criterion is not met, and the outliers need to be manually removed to yield higher registration accuracy. Further investigation is required to eliminate user intervention. For example, if a dynamic estimation method could provide additional information from the previous frames, the edges might be tracked from frame to frame to resolve this problem.
3. Coordinate system conversion. The object space coordinates system used for the 2D–3D registration is of no great significance. However, the registration and kinematics results could be converted to the anatomical coordinate system so that the motions are better described anatomically, and the registration results can be better interpreted.
4. The validation test used 3D-printed bone models to replicate the knee joint, where it may not represent the bones and soft tissue perfectly. The best option to perform the validation test is to insert beads into an in vivo knee, but it is an invasive procedure that requires the surgical implantation of markers with risks. In the future, cadaveric knees could be used for the validation test to better replicate the in vivo knee.
5. Submillimeter registration precision was achieved with the proposed method using the walking motion. In the future, different movements and conditions need to be tested, for example, lunging and standing with loads. Applications to other joints, for example, the ankle and shoulder, also need to be tested. A smaller joint or a joint with a more complex shape may affect the automatic initialization that requires more starting poses to find the approximate registration. Where real-time registration is required, e.g., image-guided interventions, machine learning can also be used for fast registration [22]. But, precision and accuracy need to be studied and compared with the current method.

## Figures and Tables

**Table 1:** Overall residuals (RMS) for the comparison of marker-based registration methods.

	RSA Method	Proposed Method	
	RMS (pix)	RMS (pix)	Improvement from RSA
Tibia	0.89	0.86	3.5%
Femur	0.89	0.85	4.7%

**Table 2:** Registration parameters with mean precision (standard deviation) of the proposed method.

	σ X (mm)	σ Y (mm)	σ Z (mm)	σ ω (°)	σ φ (°)	σ κ (°)
Tibia	±0.06	±0.11	±0.12	±0.10	±0.09	±0.09
Femur	±0.05	±0.05	±0.07	±0.08	±0.09	±0.08

**Table 3:** Registration parameters’ accuracy with rigid-body transformation for all epochs.

		X (mm)	Y (mm)	Z (mm)	ω (°)	φ (°)	κ (°)
Tibia	mean	0.32	0.02	0.11	0.34	0.04	−0.21
	σ (±)	0.27	0.26	0.48	0.63	0.28	0.39
	RMS	0.42	0.26	0.50	0.71	0.28	0.45
Femur	mean	0.02	0.00	−0.03	−0.27	−0.06	−0.01
	σ (±)	0.33	0.16	0.61	1.22	0.23	0.48
	RMS	0.33	0.16	0.61	1.25	0.24	0.49

**Table 4:** Scale factor λ estimated in each trial and the overall scale factors estimated from all the epochs.

		Trial 1	Trial 2	Trial 3	Trial 4	Overall
Tibia	mean	0.9916	0.9923	0.9921	0.9931	0.9923
	σ	0.0013	0.0015	0.0013	0.0013	0.0015
Femur	mean	0.9915	0.9932	0.9933	0.9925	0.9926
	σ	0.0010	0.0015	0.0011	0.0013	0.0014

**Table 5:** Registration parameters’ accuracy of 439 epochs with non-rigid transformation.

		X (mm)	Y (mm)	Z (mm)	ω (°)	φ (°)	κ (°)
Tibia	mean	0.01	0.15	0.11	0.25	−0.01	−0.21
	σ	0.20	0.22	0.31	0.36	0.23	0.26
	RMS	0.20	0.27	0.33	0.44	0.23	0.34
	RMS improvement	52%	4%	34%	38%	18%	24%
Femur	mean	−0.19	0.03	−0.10	−0.12	−0.03	0.02
	σ	0.18	0.13	0.32	0.52	0.23	0.23
	RMS	0.25	0.13	0.34	0.55	0.23	0.23
	RMS improvement	24%	19%	44%	56%	24%	53%

**Table 6:** The 3D reconstruction accuracy (RMS) of 439 epochs in the comparison of rigid and non-rigid transformation.

		X-Axis (mm)	Y-Axis (mm)	Z-Axis (mm)	Distance (mm)
Tibia	Rigid	0.37	0.50	0.48	0.79
	Non-rigid	0.31	0.34	0.32	0.56
	Improvement	16%	32%	33%	29%
Femur	Rigid	0.29	0.50	0.53	0.79
	Non-rigid	0.29	0.40	0.41	0.64
	Improvement	0%	20%	15%	16%

**Table 7:** The 3D reconstruction RMS error comparison of rigid and non-rigid transformations.

		X-Axis (mm)	Y-Axis (mm)	Z-Axis (mm)	Distance (mm)
Tibia	Rigid	0.52	0.50	0.58	0.93
	Non-rigid	0.38	0.37	0.42	0.68
	Improvement gained	27%	26%	28%	27%
Femur	Rigid	0.50	0.49	0.53	0.88
	Non-rigid	0.29	0.33	0.41	0.60
	Improvement gained	42%	33%	23%	32%

## Data Availability

The data are unavailable due to privacy restrictions.
